# Toy trains, loaded dice and the origin of life: dimerization on mineral surfaces under periodic drive with Gaussian inputs

**DOI:** 10.1098/rsos.170141

**Published:** 2017-11-08

**Authors:** Rowena Ball, John Brindley

**Affiliations:** 1 Mathematical Sciences Institute and Research School of Chemistry, The Australian National University, Canberra 2602, Australia; 2 School of Mathematics, University of Leeds, Leeds LS2 9JT, UK

**Keywords:** origin of life, chemical evolution, thermochemical and pH oscillator, stochastic dynamical model, left-skewed right-weighted probability distribution

## Abstract

In a major extension of previous work, we model the putative hydrothermal rock pore setting for the origin of life on Earth as a series of coupled continuous flow units (the *toy train*). Perfusing through this train are reactants that set up thermochemical and pH oscillations, and an activated nucleotide that produces monomer and dimer monophosphates. The dynamical equations that model this system are thermally self-consistent. In an innovative step that breaks some new ground, we build stochasticity of the inputs into the model. The computational results infer various constraints and conditions on, and insights into, chemical evolution and the origin of life and its physical setting: long, interconnected porous structures with longitudinal non-uniformity would have been favourable, and the ubiquitous pH dependences of biology may have been established in the prebiotic era. We demonstrate the important role of Gaussian fluctuations of the inputs in driving polymerization, evolution and diversification. In particular, we find that the probability distribution of the resulting output fluctuations is left-skewed and right-weighted (the *loaded dice*), which could favour chemical evolution towards a living RNA world. We tentatively name this distribution ‘Goldilocks’. These results also vindicate the general approach of constructing and running a simple model to learn important new information about a complex system.

## 1. Introduction

Life on the present Earth is ubiquitous, except in the most extremely arduous physical conditions; the origin of this life, however, remains an unexplained phenomenon, though available evidence seems to support its occurrence in a relatively short window of time during the period between 4.2 and 3.8 billion years ago (4.2–3.8 Ga), and at a place and under physical and chemical conditions, all of which are uncertain and virtually impossible to verify from any currently observable evidence [1,2]. There are, nevertheless, numerous clues, and in some cases circumstantial evidence, which enable us to recognize with high probability certain characteristics of the phenomenon—usually in the form of necessary (but not sufficient) requirements for its occurrence [3–5]

Though an agreed definition of life is lacking (see, for example, [6,7]) there is very wide agreement that, in the period 4.2–3.8 Ga, an evolutionary transformation occurred between non-living chemistry and living biochemistry, leading ultimately to organisms with an astonishingly resilient ability to populate the Earth we now know [8–10]. Despite the prodigious variety of living (and extinct) organisms, there is such similarity in their biochemistry and molecular biology to suggest the existence of a single last universal common ancestor (LUCA) [11,12]. There is currently a broad (but by no means universal) acceptance that, before the appearance of LUCA, prebiotic chemistry of polymerization reactions led through abiogenesis to an ‘RNA world’ from which DNA-based life ultimately evolved [13–17].

Each stage of this complex evolution demands the availability of specific chemical constituents and the existence of particular environmental conditions. The current absence of incontrovertible knowledge of either guarantees a rich field for imaginative hypotheses and consequent outcomes amenable to rigorous testing [18]. Unsurprisingly, there is a wide range of published ideas, none of which claim to be the whole truth but many of which have contributed to a broad consensus (but note [19]) on the necessary conditions and realistic processes which could have been important to life’s origin on Earth and which may have led to life elsewhere in the universe. Recent comprehensive reviews with extensive references include [20–22]. More recently, there has been a rapidly accumulating body of experimental work, both laboratory and computational, to explore plausible analogues of the actual environment of life’s origin [23,24].

In this article, we embody key aspects of this current background in presenting a model for a vital step in the evolution from chemistry to biology, based on well-supported evidence of the geophysical environment together with well-established physical and polymeric chemistry. Specifically, we take into account,

(i) the desirability of thermally cyclic environmental conditions, to achieve a ratchet effect [25] and to provide for recycling [26] of nucleotides;
(ii) the evidence for the existence at that time, and porous structure, of hydrothermal vents similar to those seen on mid-oceanic ridges at the present day [27]; and
(iii) the need to include stochasticity in any realistic model in order to represent the extreme complexity and spatio-temporal non-uniformity at both macro and micro levels of the physics and chemistry involved [28];

and conjecture that

(i) The porous structures of the vents are traversed by a high-energy, reactive flow that bears prebiotic nucleotide species. We note that, although we do not model formation of long polymers, this cellular structure with narrower interstices also permits the trapping of enhanced concentrations of longer polymers favourable for the ultimate creation of replicative RNA [29].
(ii) The relevant pre-biological chemistry takes place mainly through surface-catalysed reactions at the pore walls. There is broad acceptance that sorption and catalysis by mineral surfaces are likely to have played an essential role in facilitating the polymerization reactions that led to abiogenesis on Earth [8,30–35]. Many experimental studies report the equilibrium adsorption isotherms for biologically relevant molecules on solid surfaces (for example, [36–38] and the useful review in [32]).

This article is largely heuristic in focus and approach. Bearing in mind the considerations above, we propose a toy—but fully self-consistent—dynamical model that simulates production and dimerization of monomers from activated nucleotides in this setting. We propose that this process is effected through the thermal and pH cycling of the thiosulfate-hydrogen peroxide (THP) oscillator, as introduced originally by us in an earlier work [39] and fully characterized in [40]. (We re-present and review the THP oscillator briefly in §2.1.) In a novel extension to our earlier work, we represent the transit of a typical biomolecule through a porous matrix as a journey through a chain of interconnected continuous stirred tank reactors (CSTRs). To simulate the complexity of a real porous matrix, we build stochasticity into the model, to represent the randomly fluctuating inputs in a natural (as opposed to laboratory) setting.

We believe our simulations to be the first reported of prebiotically relevant chemistry on mineral surfaces under the strongly non-equilibrium, fluctuating, highly variable conditions that must have prevailed during the era of prebiotic chemical evolution, abiogenesis and the RNA world; thus our results strengthen the case for the essential role of mineral surface catalysis in the origin of life.

In §2.2, we adopt a simple reaction scheme, for which, importantly, experimental rate data are available, involving biologically relevant activated monomers, to provide the model prebiotic reactions to be driven by the THP oscillator. In §2.3, we present and discuss the model for a system of serially coupled, elongated rock pores, justify its use as a model for reactive flows through a porous medium, and explain the dynamical equations and computational methods. Computational results are presented and discussed in §3, and we summarize and conclude in §4. Nomenclature used in this article is given in table 1.

**Table 1.** Nomenclature, and numerical values of fixed quantities and parameters used in simulations reported in §3.

symbol (units)	definition	quantity	value
*c* or [⋯ ] (M)	concentration	—	—
$\bar{C}$ (*J* *K*^−1^ *l*^−1^)	average volumetric specific heat	$\bar{C}$	3400
*E* (kJ mol^−1^)	activation energy	—	—
*F* (μ*l* *s*^−1^)	inflow rate	*F*	0.48
*ϕ* (μ*l* *s*^−1^)	cross-flow rate	—	—
Δ*H* (kJ mol^−1^)	reaction enthalpy	—	—
*k* (*M*^−1^ *s*^−1^ or *s*^−1^)	reaction rate constant	—	—
*L* (*mW* *K*^−1^)	wall thermal conductance	*L*	2.0
*R* (*J* *mol*^−1^ *K*^−1^)	gas constant		8.31447
*T* (K)	temperature	*T*_*a*_	283
*τ* (s)	residence time	—	—
*V* (μ*l*)	unit reaction volume	*V*	10.0
*z* (*M*^−1^ *s*^−1^ or *s*^−1^)	pre-exponential factor	—	—

subscripts			
a	of the ambient or wall temperature		
*i*	of reactor unit *i*, where *i*=1…12		
*j*	of reaction *j*, where *j*=*R*1…*R*6		
*x*	a reactant or intermediate species		

## 2. Model and methods

### 2.1. The thiosulfate-hydrogen peroxide redox oscillator

The oxidation of thiosulfate by hydrogen peroxide is known to give rise to pH and thermochemical oscillations, which in a continuous-flow system are self-sustained. The reactions that have been found to contribute to this behaviour are given as follows [41–43]:

$$\begin{array}{r} {\text{H}}_{2}{\text{O}}_{2}+{\text{S}}_{2}{\text{O}}_{3}^{2-}⇌{\text{HOS}}_{2}{\text{O}}_{3}^{-}+{\text{OH}}^{-} \end{array}$$

$$\begin{array}{r} {\text{H}}_{2}{\text{O}}_{2}+{\text{HOS}}_{2}{\text{O}}_{3}^{-}\to 2{\text{HSO}}_{3}^{-}+{\text{H}}^{+} \end{array}$$

R0 $$\begin{array}{r} {\text{S}}_{2}{\text{O}}_{3}^{2-}+{\text{S}}_{2}{\text{O}}_{3}\to {\text{S}}_{4}{\text{O}}_{6}^{2-} \end{array}$$

R1, R1′ $$\begin{array}{r} {\text{H}}_{2}\text{O}⇌{\text{H}}^{+}+{\text{OH}}^{-} \end{array}$$

$$\begin{array}{r} {\text{H}}_{2}{\text{O}}_{2}+{\text{HSO}}_{3}^{-}\to {\text{SO}}_{4}^{2-}+{\text{H}}_{2}\text{O}+{\text{H}}^{+} \end{array}$$

$$\begin{array}{r} {\text{H}}_{2}{\text{O}}_{2}+{\text{HSO}}_{3}^{-}+{\text{H}}^{+}\to {\text{SO}}_{4}^{2-}+{\text{H}}_{2}\text{O}+2{\text{H}}^{+} \end{array}$$

R2, R2′ $$\begin{array}{r} {\text{HSO}}_{3}^{-}⇌{\text{H}}^{+}+{\text{SO}}_{3}^{2-} \end{array}$$

$$\begin{array}{r} {\text{HOS}}_{2}{\text{O}}_{3}^{-}+{\text{H}}^{+}⇌{\text{S}}_{2}{\text{O}}_{3}+{\text{H}}_{2}\text{O} \end{array}$$

R3 $$\begin{array}{r} {\text{S}}_{2}{\text{O}}_{3}^{2-}+2{\text{H}}_{2}{\text{O}}_{2}\to \frac{1}{2}{\text{S}}_{3}{\text{O}}_{6}^{2-}+\frac{1}{2}{\text{SO}}_{4}^{2-}+2{\text{H}}_{2}\text{O}. \end{array}$$

As elucidated in [40], at low concentrations of the feed reactants (∼mM) this system behaves as a nearly isothermal pH oscillator, with production of tetrathionate in reaction R0. At relatively high input concentrations (∼M) of hydrogen peroxide and thiosulfate, R0 and all the unlabelled reactions are suppressed and a deep, highly exothermic oxidation takes over, with production of trithionate in reaction R3. Under these conditions, the system exhibits strong thermochemical oscillations, which are accompanied, in the presence of the acid–base equilibria R1,*R*1′ and R2,*R*2′, by pH oscillations.

In [40], we unified the isothermal pH and thermochemical-pH regimes of the THP oscillator smoothly and self-consistently for the first time, and showed in dynamical simulations of a CSTR configuration that it can drive pH-dependent replication of a short RNA species by complementary strand pairing. In this work, we exploit the thermochemical-pH-oscillatory regime, thus we retained only reactions R3, R2,*R*2′ and R1,*R*1′ in our simulations. Numerical values of the rate parameters and reaction enthalpies are given in table 2.

**Table 2.** Experimental values of the thermokinetic parameters and reaction enthalpies for reactions R1–R6, from [41–44].

reaction	*z*(*M^−1^ s^−1^)(**s^−1^)	*E* (kJ mol^−1^)	Δ*H* (kJ mol^−1^)
R1	4.55×10^6^**	55.84	55.84
R1′	1.32×10^9^*	0	−55.84
R2	3.95×10^16^**	75.1	49.3
R2′	1.61×10^15^*	25.8	−49.3
R3	1.63×10^10^*	68.12	−572.2
R4	1.0×10^6^**	57.0	n.a.
R5	17.5*	27.0	n.a.
R6	6.8×10^7^**	100	n.a.

For this work, we take the availability of a continuous supply of the THP reactants as given. Compendiums of the literature on the sources and occurrence of non-biologically produced hydrogen peroxide and thiosulfate on the Hadean and early Archean Earth, and throughout and beyond the solar system, are given in [39,40].

### 2.2. Prebiotic reactions

For the model prebiotic reactions to be driven by the THP oscillator we chose the montmorillonite-catalysed hydrolysis of the activated 5′-monophosphorimidazolide moiety of cytidine (ImpC), to cytidine 5′monophosphate (pC), followed by dimerization and degradation of the dimer. These reactions were studied experimentally by Kawamura & Maeda [44], and this is one of the few prebiotically relevant reaction systems for which the thermokinetic parameters have been determined over the temperature range of the THP oscillator. The reactions are given as follows:

R4 $$\text{ImpC}\overset{k_{\mathrm{hy}}}{⟶}\text{pC},$$

R5 $$\text{ImpC}+\text{pC}\overset{k_{\mathrm{di}}}{⟶}\text{dimer},$$

R6 $$\text{dimer}\overset{k_{\text{hy,}2}}{⟶}\text{products}.$$

Reaction R4 represents adsorption to the montmorillonite surface and hydrolysis to the monophosphate in a single process. Reactions R5 and R6 take place on the montmorillonite surface; products are released into the CSTR unit stream. Numerical values of the rate parameters are given in table 2. (As described in §2.3, the enthalpies for these reactions are negligible in the context.)

### 2.3. Pore model

The physical system we would like to model is a flow through an elongated rock pore, or train of serially coupled rock pores. Although any real porous rock matrix, such as those around the types of submarine hydrothermal vents that have been postulated as settings for the origin of life [8], is of course three dimensional, the experience of a prebiotic molecule carried in the flow is that of passing through a succession of pores connected by narrower interstices. In that sense its trajectory is effectively one dimensional, and therefore we have chosen to model the rock pores as a train of continuous flow, well-stirred reactors (CSTRs) coupled in series. CSTRs-in-series models have been used mainly in chemical engineering contexts (for example, [45]), but this is not without precedent in principle, if not in practice, in the current context: Czárán *et al.* [35] invoked a series-coupled stirred tank reactor paradigm conceptually, in the context of a cellular automaton model for an isothermal replicating system in a porous rock matrix.

**Figure 1.**
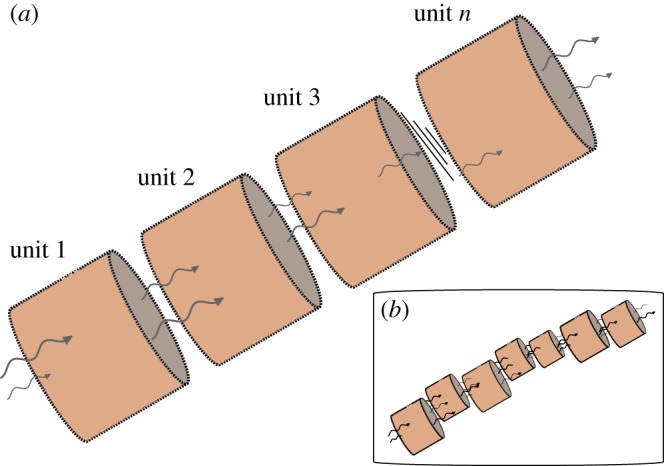
(*a*) Sketch of the CSTRs-in-series set-up in which *n* units are coupled serially. THP reactants flow in at one end; dotted outlines indicate porous walls through which THP reactants also enter the system in cross-flow. The cross-flow mean residence times may differ between units, which allows the model to account for the more general situation shown in (*b*), where the pore volumes are non-uniform.

The set-up is sketched in figure 1*a*. Reactants for the THP oscillator (thiosulfate, hydrogen peroxide and acid) and ImpC are infused continuously at one end. In addition, we assume that the walls of each unit are porous to the THP reactants, hence allowing them to be replenished continuously along the system, although the walls are impervious to the larger species of reactions R5–R7. In this aspect, our ‘toy train’ is analogous to the cross-flow reactor, a tube with a longitudinal porous membrane through which reactants can be supplied continuously and selectively, which is studied in chemical engineering contexts (for example, [46]). The advantages of the CSTRs-in-series model over a tubular reactor model in the current context will be apparent in §3.1, where we report results that simulate wall inhomogeneities.

Mass and enthalpy balances yield coupled differential equations for evolution of the temperature and concentrations of species in reactions R1,*R*1′, R2,*R*2′, R3, and R4, R5 and R6 at the outlet of each unit in figure 1*a*.

The dynamical equation for the temperature in unit *i*,

2.1 $$V\bar{C}\frac{\text{d}T_{i}}{\text{d}t}=V\sum_{p}(-\Delta H_{j})r_{j,i}(T)+\bar{C}(\tilde{F}T_{i-1}-\tilde{F}T_{i})+\bar{C}({\tilde{\phi }}_{i}{\tilde{T}}_{a}-{\tilde{\phi }}_{i}T_{i})+L({\tilde{T}}_{a}-T_{i}),$$

includes the following contributions on the right-hand side: The first term is the total rate of heating due to reaction enthalpies, where *r*_*j*,*i*_(*T*) is the rate of reaction *j* in unit *i* and the summation is over the *p* reactions that contribute to the enthalpy. The second and third terms account for the heat transported by the inflow and cross-flows, respectively. The fourth term is the rate of heat loss to an environment that is assumed to remain at a fixed ambient temperature *T*_*a*_.

Mass balances in each unit *i* yield dynamical equations for the concentrations,

2.2 $$V\frac{\text{d}c_{x,i}}{\text{d}t}=V\sum_{m}\sigma r_{j,i}(T)+\tilde{F}c_{x,i-1}-\tilde{F}c_{x,i}+{\tilde{\phi }}_{i}{\tilde{c}}_{x,a}-{\tilde{\phi }}_{i}c_{x,i},$$

where *c*_*x*,*i*_ is the concentration of reactant or intermediate *x* in unit *i*, the summation is over the *m* reaction rates that involve *x*, and *σ*=1 for intermediates that are produced and *σ*=−1 for reactants and intermediates that are consumed. The rate coefficients *k*_*j*,*i*_ of the reaction rates *r*_*j*,*i*_ have Arrhenius temperature dependence,

2.3 $$k_{j,i}(T)=z_{j}\exp\left(\frac{-E_{j}}{RT_{i}}\right).$$

(In §3.2, the rate constants for reactions R4 and R5 also will be given specific pH dependences.) The symbols in equations (2.2)–(2.4) are defined in table 1, where numerical values are given for quantities that are constants and those that are fixed across all simulations.

Some of the qualities of and assumptions built into equations (2.2) and (2.3) are highlighted and clarified as follows.

— The usual CSTR assumptions and approximations apply, so we do not enumerate them here. They are well known in the chemical engineering community; for others an accessible reference is [47]. (The original authority on continuous-flow systems is Denbigh [48], who, rather presciently, draws attention to their potential usefulness in imitating biological processes.) The possible effects of inclusion of diffusion and convection, i.e. relaxation of the well-stirred assumption, were canvassed in [40].
— A novel feature is that the input parameters are subject to stochastic fluctuations, which we have modelled by treating the inflow rate, cross-flow rates, input concentrations and ambient temperature as normally distributed random quantities $\tilde{q}=\bar{q}+\delta q$, where $\bar{q}$ is the mean, *δq* is the random fluctuation and the variance $s=w\bar{q}$. After numerical experimentation, we found that *w*=0.02 gave a satisfactory response, i.e. illustrated the effects of stochasticity while not being unrealistic in a dynamic, non-equilibrium setting, and not imposing a too formidable computational burden.
— We have to accommodate the fact that a rock pore is not a precisely machined tube in a chemical engineering laboratory. The walls are non-uniform in composition and thickness, and present spatially variable resistance to cross-flows. To model this heterogeneity, each mean cross-flow coefficient $\bar{\phi_{i}}$ is independently variable in the implementation of the model.
— The volumes of the units are considered identical. This does not affect the generality of the model, because the residence times ${\tilde{\tau }}_{i}=V/(\tilde{F}+{\tilde{\phi }}_{i})$ may differ between units, as suggested in figure 1.
— In implementing this model, we found that setting *n*=12 for the number of coupled units was a good trade-off between computational convenience (i.e. integration times on a laptop computer of ⪅10 min for a typical run, noting that many hundreds of trial, optimization and equilibration runs had to be made before final runs and data collection) and illustrating the effects of variable residence times and stochasticity and obtaining significant reactant conversion.
— Obviously for unit 1, $T_{i-1}\equiv {\tilde{T}}_{a}$ and $c_{x,i-1}\equiv {\tilde{c}}_{x,a}$.
— As mentioned earlier, we assume that ImpC enters the train at the inlet to unit 1, but the walls are impervious to ImpC, pC, dimer and the degradation products in reactions R4–R6. Therefore, in the species balances for these reactants (equation (2.3)) the ${\tilde{\phi }}_{i}=0$.
— The *specific* reaction enthalpies of reactions R4–R6 are negligible, because the concentrations are substantially lower than those of the THP reactants. In practice, we found that including enthalpy generation terms for these reactions in the enthalpy balance, equation (2.2), made negligible difference to the output, hence for the sake of minimizing run times, we omitted them. However, reactions R4–R6 are still coupled to the temperature via equation (2.4).

The model we have constructed with equations (2.2) and (2.3) is very much a toy representation of a system of reactive flows through a porous maze. Like all toys it is scaled down, vastly simplified, neglects many features of the real system while exaggerating others (e.g. we have increased the inflow concentration of ImpC in order to obtain significant amounts of pC and dimer within a train of 12 units) and easily broken but quickly fixed—i.e. modular. We believe there is much to be learned about a complex system by studying such models, and this will be borne out by our results in §3. The model is not a trifle, though, because it includes (i) the dynamic coupling of temperature and concentration self-consistently and its outputs therefore can document the phenomenology of the THP oscillator and its interactions with prebiotically relevant molecular species in an extended system; and (ii) stochastic inputs, which produce non-intuitive results that may be of profound significance to our understanding of the origin of life (§3.3).

Equations (2.2) and (2.3) were solved numerically using XPPAUT [49]. For each run, equations (2.2) and (2.3) were integrated from convenient initial conditions using a stiff integrator until the trajectory settled reliably on the stable limit cycle attractor, usually for approximately 6000 *s*. Integration was then continued for 3000 s or more and this time series was captured. For the results in §3.3 very long time series approximately 40 000 *s* were computed, and the data aggregated over the 12 units. Numerical values of the fixed quantities and parameters of the model given in tables 1 and 2 were used.

The raw data were exported to Excel spreadsheets for post-processing. Accurate, weighted averages of oscillating variables were computed by integrating over the time window using the trapezoidal rule, and dividing by the number of seconds in the window (namely, 50 000 s in each case). This is necessary because the oscillations are not purely sinusoidal. We denote quantities averaged in this way as ‘int-av’.

## 3. Results and discussions

In §§3.1 and 3.2, we inspect and discuss the outputs where reactions R4 and R5 are pH-independent and pH-dependent, respectively.

In §3.3, we exploit the stochasticity of the inputs to interrogate the distribution of the output temperature fluctuations, and obtain a result that leads to a profound insight on necessary conditions for the origin of life and suggests a new test diagnostic for identifying potential habitats for extraterrestrial life.

### 3.1. pH-independent reactions R4 and R5

**Figure 2.**
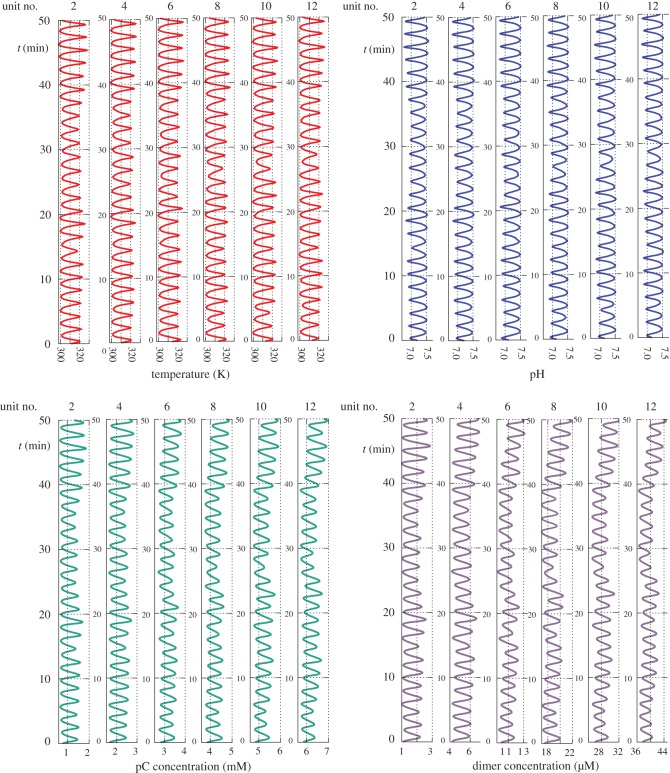
Time series where the cross-flow rates are identical, ${\bar{\phi }}_{i=1..12}=4.8\;\text{μ}{l\;s}^{-1}$. Owing to the stochastic fluctuations, the temperature and pH oscillations vary in period and amplitude within and between units, but there is no overall trend. Note the increasing concentrations of pC and dimer along the train, and over time.

For this case, we will compare outputs for three longitudinal profiles of the mean cross-flow rates $\bar{\phi }$. But first let us inspect the raw time-series outputs for a flat profile, where the ${\bar{\phi }}_{i}$ are identical for all units. In figure 2, the time series are rendered in terms of the temperature, pH, and pC and dimer concentrations, in alternate units (for conciseness of the figure).

The oscillations appear deterministically chaotic but they are not; the non-uniformity of period and amplitude is simply due to the randomly fluctuating inputs. This is satisfying, because the ability to sample more extreme conditions occasionally is an advantage in a world evolving towards assembly of a diverse range of prebiotic polymers. This stochasticity of the outputs will be analysed in §3.3.

Note in figure 2 that the pC and dimer concentrations steadily increase along the train. This suggests that prebiotic polymerization reactions would have been favoured by highly elongated systems of rock pores. Thus, in a longer, more expensive ‘toy train’ of perhaps hundreds of units, we would expect trimers and longer polymers to appear, assuming these species can be included in the *c*_*x*,*i*_ of equation (2.3) and the extra rate data are available.

**Figure 3.**
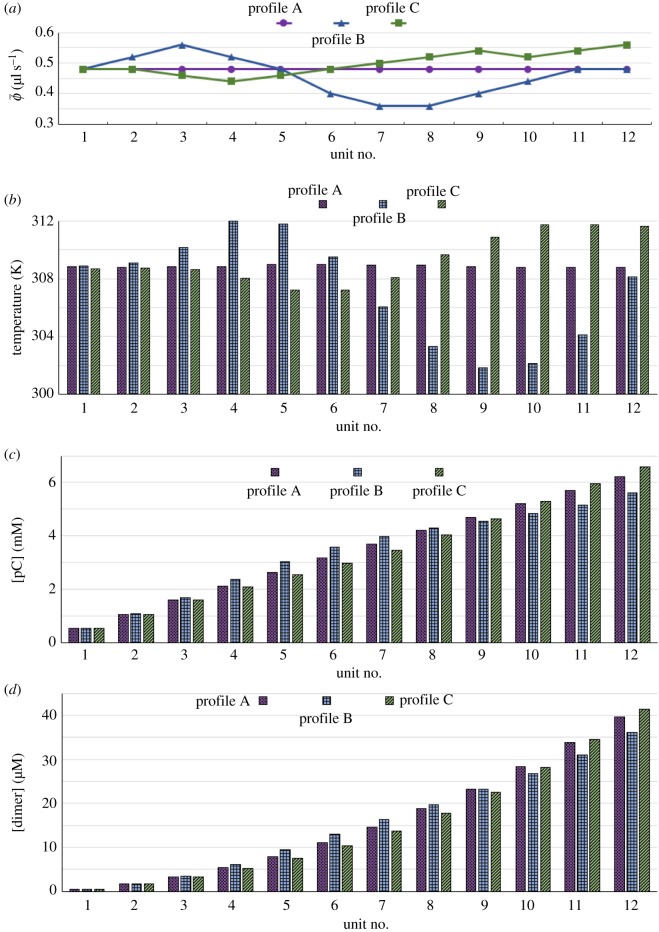
The effects of non-uniform cross-flows along the length of the train. The temperatures and pC and dimer concentrations are int-av quantities.

The three longitudinal profiles of $\bar{\phi }$ for which we compare outputs are shown in figure 3*a*. (For heuristic purposes we have smoothed over the discrete profiles.) The effects of profiles B and C on the temperatures and on pC and dimer production can be read off the clustered column charts of figure 3*b*, *c* and *d*, where these have been computed as int-av quantities, as described at the end of §2.3.

The initially increasing $\bar{\phi }$ of profile B sees the temperature increasing (*b*), but there is a finite-time effect in that the temperature maximum at unit 4 lags the $\bar{\phi }$ maximum at unit 3. This is because, as the mean residence times of the THP reactants increase after the $\bar{\phi }$ maximum, more heat is produced by the exothermic reactions. As the ${\bar{\phi }}_{i}$ decreases along profile B, the temperature drops steeply, but again the minimum temperature at unit 9 lags the minimum $\bar{\phi }$ at units 7–8—in this case it is reactant supply that is limiting. The lag persists, so that when $\bar{\phi }$ in units 11 and 12 is again equal to its value in unit 1, the temperature has not regained its value in unit 1.

We observe this finite-time effect again in profile C, where the temperature drop lags the initial decline of $\bar{\phi }$, the temperature maximum lags that of ${\bar{\phi }}_{i}$, and in unit 6 the temperature has not recovered to that in units 1 and 2. This inertia is also apparent in units 9–11, where the temperature does not ‘see’ the wobble in $\bar{\phi }$.

What are the effects of these non-uniform $\bar{\phi }$ profiles on pC and dimer production? The profiles affect reactions R4 and R5 through their influence on the temperature. This can be elicited qualitatively by inspection of figure 3. At first the higher temperatures induced by profile B increase the concentrations of pC (*c*) and dimer (*d*) relative to profile A, but by unit 9 this is reversed. The decreasing temperatures due to profile C inhibit the reactions, relative to profile A, up to unit 9, but by unit 10 the concentrations are higher than those for profile A.

**Figure 4.**
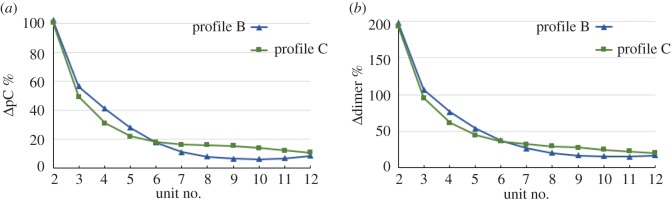
The percentage increases between adjacent units of (*a*) pC and (*b*) dimer, for profiles B and C in figure 3*a*.

A quantitative comparison of the non-uniform profiles on reactant conversion is given in figure 4, where the percentage increases of pC (*a*) and dimer (*b*) between adjacent units are plotted. The percentage increases fall more steeply for profile C than for profile B at first, because the temperatures are lower. The crossover point at unit 6—where the temperature for profile B is higher than that for profile C—belies the effects of the residence times on reactant availability.

In summary, the pore profile inhomogeneities effectively broaden the range of temperatures and residence times that the system may sample and thus can change the distribution of products along the profile. For a prebiotic chemical world, evolving towards a living world, this would provide significant advantages. A homogeneous profile (profile A in figure 3*a*) gives the system very little capacity to ‘try out’ or reject alternative reactions, in other words, to grow and morph.

In general we see, too, why for this purpose a CSTRs-in-series model is superior to a tubular reactor model with cross-flow [46]: the tubular reactor model must have a longitudinally homogeneous cross-flow.

So we want our rock pore crucible of life to be inhomogeneous. But not *too* inhomogeneous! The large finite-time lag effects of gross inhomogeneities would lead to dramatic temperature changes persisting downstream, more probably extinguishing a precious synthetic reaction or damaging a catalytic surface than enabling growth and diversification.

### 3.2. pH-dependent reactions R4 and R5

Kawamura & Maeda [44] carried out their experiments at a constant pH of 8. However, it is well known that the pH of the medium can have strong effects on surface reactions, because the pH affects the charge status of, and hence the electrostatic interactions between adsorbent and adsorbate. Our system has a ready-made, self-consistently varying pH (figure 2), so we can exploit this in a natural manner if we know the pH dependences of the rate constants of reactions R4 and R5. Aldersley *et al.* [50] studied the adsorption and polymerization of activated nucleotides on montmorillonite. From their experimental data, we have fitted the pH dependences of reactions R4 and R5, over our pH range of interest, as follows:

3.1 $$k_{\mathrm{hy}}^{\prime }=\frac{8k_{\mathrm{hy}}}{\text{pH}}$$

and

3.2 $$k_{\mathrm{di}}^{\prime }=\frac{k_{\mathrm{di}}{\text{pH}}^{1/2}}{8^{1/2}}.$$

(For heuristic purposes reaction R6 remains pH-independent.) Equations (3.1) and (3.2) say that increasing the pH decreases the rate of reaction R4 and increases the rate of reaction R5.

**Figure 5.**
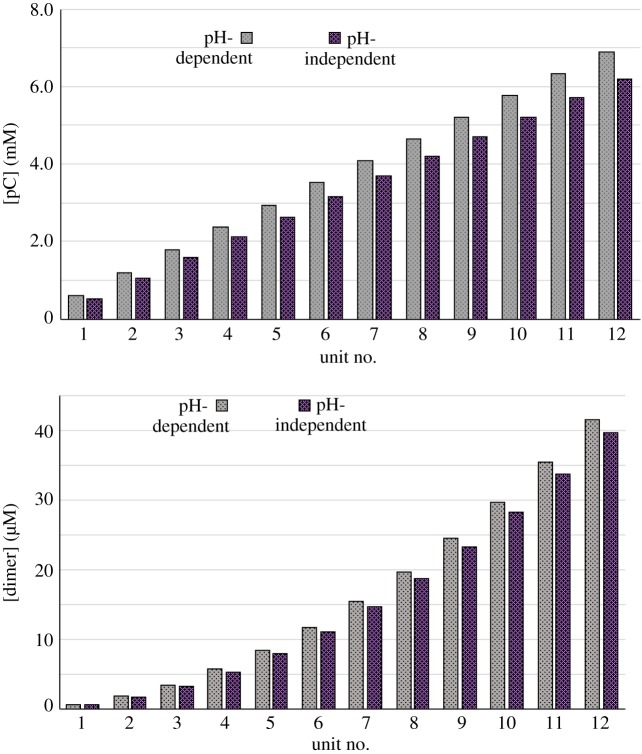
Results for reactions R4 and R5, where the rate constants have the pH dependences given in equations (3.1) and (3.2), are compared with the pH-independent case.

We carried out simulations for profile A in figure 3*a*, incorporating the dependences (3.1) and (3.2), and compared the outputs with those for the pH-independent case. At the int-av pH of 7.27 the rate of reaction R4 is 1.10 times that of the pH-independent case, and the rate of reaction R5 is 0.95 times that of the pH-independent case. However, as it is the availability of pC that is limiting, due to the higher activation energy of R4, the overall effect is to enhance both pC and dimer production, relative to the pH-independent case. This result is illustrated in figure 5.

In the heuristic spirit of this paper, we can use this result to make some inferences about a prebiotic world, chemically evolving towards the RNA world, under the drive of the THP oscillator. First, pH-dependent reactions and processes are likely to have been selected for. Second, in the presence of a constant pH only a very narrow range of pH-dependent reactions could occur. Third, it follows that a periodically varying pH would enable a wider variety of reactions and facilitate growth and change in the system.

So we want the pH to be variable. But not *too* variable! A system that spends significant time at pH extremes is more likely to hydrolyse, or collapse into hopeless tars, than grow and polymerize and morph.

### 3.3. Random is as random does

In the chemical engineering laboratory or production facility, the effect of normally distributed random input fluctuations inevitably weakens ‘performance’, which in that context is defined in terms of some index of process intensification, usually maximizing the yield of a single product and minimizing side reactions and back reactions. That is why chemical engineers seek to design streamlined processes with minimal perturbations. In an origin of life context, though, ‘performance’ must be defined in terms of potential of a prebiotic chemical system to grow, evolve and diversify. In that case, what are the effects of normally distributed, random perturbations of the inputs on the outputs of the system?

The following finding, by Bertola & Cafaro [51], turns out to have crucial importance in an origin- of-life context:

(A) *Gaussian perturbations through the boundary of a system do not induce Gaussian fluctuations in the outputs.*

This fact is not self-evident *a priori*, but it was confirmed rigorously by Bertola & Cafaro [51]. They modelled the enthalpy evolution of a simple, non-reactive, closed thermodynamic system, close to equilibrium but perturbed by a Gaussian stochastic heat input through the boundary. They found that the resulting temperature and entropy fluctuations are described by a gamma probability density function.

Like that analysed by Bertola & Cafaro, our system, equations (2.2)–(2.4), is subject to Gaussian input perturbations, but there are substantial differences too: ours is open, reactive, strongly nonlinear and far from equilibrium. Although not amenable to analytic study, we can test (A) in our system numerically. Specifically, we interrogate the fluctuations produced in the temperature maxima (figure 2), ${\bar{T}}_{\max}-T_{\max}$. We shall see that (A) holds, but a gamma-type distribution of the fluctuations is actually antithetical to the origin of life.

**Figure 6.**
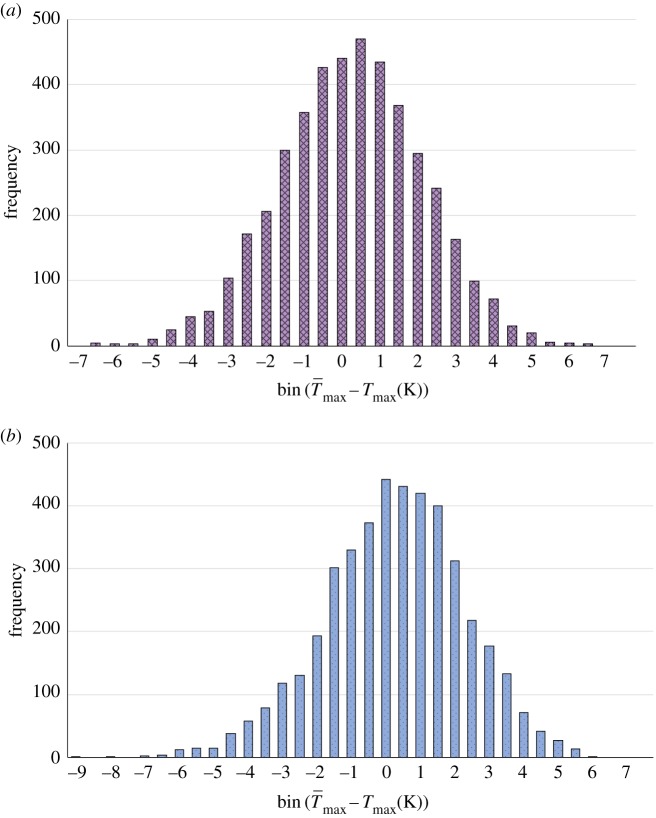
Histograms of perturbations of the temperature oscillation maxima, where the rates of reactions R4 and R5 are (*a*) pH-independent and (*b*) pH-dependent as given by equations (3.1) and (3.2). (These simulations use profile A in figure 3*a*.)

We carried out statistical analysis of data from pH-independent and pH-dependent simulations, using profile A in figure 3*a*, and found the negatively skewed (tail on the left side is longer than the right side), right-weighted distributions of perturbations to the temperature maxima shown as histograms in figure 6, and given in the appendix, table 5. (Only genuine maxima were included in the datasets. Spurious maxima, which are inevitably present in the raw data due to the random perturbation at each time step, were thoroughly and painstakingly screened out.) The summary statistics given in table 3 describe the departure of the distributions from normal. The median *M* shows there is a small—but significant—weight of higher temperature fluctuations (the mean is essentially zero). The negative skewness *S* shows that more low-temperature bins are populated than high-temperature bins. The positive kurtosis *κ* tells us that the tails are heavier than those of a normal distribution.

**Table 3.** Summary statistics for the distributions in figure 6: median *M*, skewness *S*, and kurtosis *κ*. (The kurtosis is given as calculated by Excel, which defines the kurtosis of a normal distribution as equal to zero.)

*M*	0.024	0.090
*S*	−0.08	−0.27
*κ*	0.02	0.19

A gamma distribution has positive skewness and is left-weighted. In thermodynamic systems, in the form of the Maxwell–Boltzmann distribution, it describes the distribution of molecular speeds at a particular temperature and applies to the realm of dilute ideal gases at equilibrium. So it is not surprising (*a posteriori*, at any rate) that Bertola & Cafaro’s fluctuations exhibit a gamma distribution. Evidently, the addition of mass flows, strong nonlinearity, and a dynamic, far-from-equilibrium drive—all necessary (but not sufficient) conditions for life—changes the distribution profoundly.

There are several notable aspects to these results, which we enumerate in the following discussion.

(i) First, we note that the presence of fluctuations *in itself* is advantageous for a growing, diversifying prebiotic system. For a profile A, pH-independent simulation with zero input perturbations, the maximum temperature of each cycle is frozen at 325.0 K. The average maximum temperature of the cycles when random input perturbations are switched on is 322.5 K, but such a system can sample a wide range of extrema, as is evident in figure 6*a*, giving it greater capability to produce a variety of molecular products from reactions having different activation energies. The fluctuations that weaken the performance of the chemical engineering facility actually *strengthen* that of a prebiotic system.
(ii) The positive median and negative skewness of both distributions in figure 6 show that the systems are weighted towards higher temperature perturbations, thus reactions of higher activation energy are relatively favoured over those of lower activation energy. Our simple model allows us to test this supposition by comparing conversion data under non-fluctuating and fluctuating inputs for reactions R4 (relatively high activation energy) and R5 (relatively low activation energy).
  We expect that int-av product yields inevitably will be lower under fluctuating inputs, but the right-weighted distributions of output fluctuations should favour high activation energy reactions over low activation energy reactions. This is borne out by the data analyses presented in table 4. As expected, fluctuating inputs lead to lower output concentrations of both products. But the product from the higher activation energy reaction, pC, is suppressed to a lesser extent than that from the lower activation energy reaction, the dimer. The precious, high activation energy synthetic reactions that the prebiotic, non-enzymic world *must* access in order to grow, polymerize and diversify are enabled by a negatively skewed, right-weighted distribution of temperature fluctuations!
(iii) It becomes apparent, too, why a gamma distribution simply won’t do for the origin of life. Under a gamma distribution of output temperature fluctuations, a high activation energy reaction would undergo its low activation energy reverse with higher probability, and development of a more complex prebiotic world would be impossible. Even worse, such a left-weighted, right-skewed distribution would favour degeneration to a *simpler* system with no capacity to evolve.
  The values for the summary statistics in table 3 seem ‘small’, but we found them to be robust with respect to numbers of data points and data window over which they are computed. The median and skewness of the pH-dependent distribution (figure 6*b*) are more than thrice, and the kurtosis more than nine times that of the pH-independent distribution (figure 6*a*), hence making use of the pH oscillations to drive pH-dependent reactions confers a great advantage on a developing prebiotic world, in terms of being able to access higher temperatures more often.
  But not too often and not too high! The median and skewness and kurtosis must not be too large! There would be optimum values such that the system spends enough time at higher temperatures to carry out essential high activation energy reactions, but not so much that the reactant is totally consumed, or destroyed, or surfaces degraded. For this reason we tentatively name the distribution ‘Goldilocks’.

**Table 4.** Comparison of product yields in unit 12 from reactions R4 (relatively high activation energy) and R5 (relatively low activation energy) under non-fluctuating and fluctuating inputs, for pH-independent and pH-dependent cases.

inputs		[pC]_12_ (mM)	[dimer]_12_ (μM)
pH-independent			
non-fluctuating		6.44	41.2
fluctuating		6.20	39.6
	%change	-3.75	-3.89
pH-dependent			
non-fluctuating		7.20	43.5
fluctuating		6.88	41.5
	%change	-4.46	-4.51

Although there is a body of work in the literature that is concerned with the role of stochasticity in the origin of life, this is still largely a subject awaiting investigations. Higgs & Wu [28] treat the origin of the living state itself as a rare, stochastic event. They carried out stochastic simulations of simple diffusing replicators on a lattice and found that a rare, localized large concentration fluctuation can transition the non-living state to the living state in a localized region, which can then spread rapidly.

Stochastic fluctuations also have been invoked in explanations for the origin of biological homochirality [52], although their role has been disputed [53]. In established life, random variation is, of course, the keystone of Darwinian evolution, and this is implicit in much of the literature dealing with evolution of the RNA world (see review [22]).

Szostak [54] hypothesized that a messier, more complex environment and set of substrates than the usual laboratory experimental set-up—which implies greater stochasticity—may actually facilitate prebiotic processes: ‘Scenarios involving moderate chemical and physical complexity are, in general, more geochemically sensible, and thus more prebiotically plausible than over-simplified laboratory models. It would, therefore, be very satisfying indeed if such scenarios turned out to be not only compatible with but also necessary for the key steps in the chemical origins of life.’ Our results strongly support this hypothesis.

We can now suggest the following corollary to (A), as it pertains to the origin of life:

(B) *For life to originate the habitat must exhibit a Goldilocks distribution of temperature fluctuations.*

Characterizing this distribution in explicit form, and its parameters and moments (obtaining the fundamental equation of life!), is a project for the future, but meanwhile our results have placed stochasticity explicitly at centre stage in the origin-of-life drama. The importance of fluctuations is expressed succintly by the title of this subsection, which idiomatically is a way of saying that a system subject to input perturbations will act according to its distribution of output perturbations.

The dice on the primordial Earth were loaded in favour of the emergence of life. In the search for extraterrestrial life, the traditional strategy is ‘Follow the water’. Given (B), we suggest that an additional new strategy might be ‘Sample the temperature fluctuations’. Applying this diagnostic (using, we would imagine, rather cheap spectroscopic methods) to extraterrestrial potential habitats could easily eliminate unlikely ones—those that are not sufficiently out of equilibrium to have any hope of supporting life or incipient life and those for which the temperature fluctuations are too extreme—and we need not waste further time or money on them.

## 4. Conclusions

(i) Outputs from a toy model for prebiotic reactions driven by the redox THP oscillator in a hydrothermal rock pore have yielded novel and genuine insights into and understanding of the truly ‘wicked’ problem of the origin of life on Earth.
(ii) Although there is broad (but not universal) acceptance of a hydrothermal rock pore setting for the origin of life, knowledge of the specifics of the structure is lacking. Our simulations have elicited the importance of *long* rock pores, or *lengthy, interconnected* porous structures in enabling synthesis of long polymers. In other words, the residence times of prebiotic monomers and short polymers must be long enough to allow sequential reactions to produce longer polymers. A simple observation, this, but if we wish to localize the origin of life on Earth, it does provide useful guidance. The tall, hydrothermal chimneys favoured by Russell & Hall [55] and others seem likely settings by this criterion, whereas the small crater lakes favoured by Sankar [56] do not.
(iii) Prebiotic molecular growth and evolution are favoured by non-uniformity of the rock pore walls, which broadens the range of temperatures and residence times that prebiotic molecular species will experience, due to the lag effect, and thus increases the range of reaction paths available. Note that it is a toy property of the model that elicits this insight—namely, allowing variability of the cross-flow rates as proxy for wall inhomogeneity.
(iv) Simulations incorporating the pH-dependence of reactions R4 and R5 show enhanced production of pC and dimer. The highly pH-responsive operation of all biology we know of, the universal dependence of life on pH gradients, is likely to have been selected for at the prebiotic stage.
(v) It is a profound insight, and perhaps a profound anticlimax (or even a profound existential put-down, given our anthropocentric vanities) that life on Earth may owe its existence to a left-skewed, right-weighted probability distribution of temperature oscillation maxima. Its effects are highly life-favouring, enabling the precious, high activation energy reactions that lead to polymerization, diversification and growth in a non-enzymatic world.

## Supplementary Material

Figure 5 data

## Supplementary Material

Figures 3 and 4 data

## Supplementary Material

Toy Train

## Data accessibility

The code used to generate the data and the data files themselves are available as online electronic supplementary material.

## Appendix A

**Table 5.** Data for histograms in figure 6. For each distribution the total number of points is 4353.

	frequencies	
bin	pH-independent	pH-dependent
−9	0	1
−8.5	0	0
−8	0	1
−7.5	0	0
−7	0	2
−6.5	4	4
−6	3	12
−5.5	3	15
−5	10	15
−4.5	25	38
−4	45	58
−3.5	53	79
−3	104	118
−2.5	172	130
−2	206	193
−1.5	300	301
−1	357	330
−0.5	426	373
0	441	420
0.5	470	431
1	434	420
1.5	368	400
2	295	312
2.5	241	217
3	163	177
3.5	99	133
4	72	71
4.5	31	41
5	20	27
5.5	5	13
6	4	1
6.5	3	0
7	0	0
